# AI virtual streamers in sustainable food marketing: how expertise, interactivity, and anthropomorphism shape consumer acceptance of upcycled foods

**DOI:** 10.3389/fnut.2026.1903565

**Published:** 2026-09-09

**Authors:** Liang Shan, Yunhao Zhang, Yuguang Ma, Xiaoya Chen, Youyou Li, Jing Tian

**Affiliations:** 1 Network Information Center, Tonghua Normal University, Tonghua, Jilin, China; 2 School of Mathematics and Artificial Intelligence, Tonghua Normal University, Tonghua, Jilin, China; 3 Department of Business Administration, Dongshin University, Naju-si, Jeollanam-do, Republic of Korea; 4 Institute of International Economics and Trade, Liaoning University of International Business and Economics, Dalian, Liaoning, China; 5 School of Pharmacy and Health Sciences, Tonghua Normal University, Tonghua, Jilin, China

**Keywords:** artificial intelligence, perceived value, product trust, purchase intention, upcycled foods, virtual streamer

## Abstract

**Purpose:**

Drawing on the stimulus–organism–response (SOR) framework, this study examines how AI virtual streamer expertise, interactivity, and anthropomorphism influence consumers’ purchase intention through perceived value and product trust.

**Methods:**

A three-wave time-lagged survey was conducted among adult consumers in China. After data screening and anonymous matching across the three waves, 392 valid responses were retained. Structural equation modeling and bootstrap analysis were used to test the hypothesized relationships and indirect effects.

**Results:**

Expertise and interactivity were positively associated with both perceived value and product trust. Anthropomorphism was positively associated with product trust, but its relationship with perceived value was not significant. Perceived value and product trust both positively predicted purchase intention. The mediation analysis showed that perceived value mediated the relationships of expertise and interactivity with purchase intention, but did not mediate the effect of anthropomorphism. Product trust mediated the relationships between all three AI virtual streamer characteristics and purchase intention.

**Conclusion:**

AI virtual streamer characteristics influence consumer acceptance of upcycled foods through different psychological pathways. Expertise and interactivity strengthen both perceived value and product trust, whereas anthropomorphism mainly supports trust formation. These findings clarify the distinct roles of AI communication cues and highlight the importance of combining clear product information, responsive interaction, and trust-building strategies in sustainable food marketing.

## 1 Introduction

Food waste has become a persistent challenge in the transition toward more sustainable food systems. Large quantities of edible materials and food-processing by-products continue to be discarded, underused, or converted into low-value outputs across production, processing, distribution, and consumption (1, 2). Upcycled foods offer a potential response to this problem by transforming ingredients that might otherwise be wasted or undervalued into new food products through processing innovation, formulation improvement, and value enhancement (3). Examples include snack bars made from fruit pomace, bread or biscuits containing flour derived from spent grain, beverages produced from fruit and vegetable processing by-products, and foods made from edible produce that does not meet conventional appearance standards. Unlike conventional waste-reduction practices, food upcycling combines resource recovery with the creation of new nutritional, functional, and economic value, making it relevant to both circular food systems and sustainable consumption (4).

The sustainability benefits of upcycled foods, however, do not automatically lead to consumer acceptance. Consumers may associate these products with food waste reduction, resource conservation, and responsible consumption (5), but they may also question where the ingredients come from, how intensively they are processed, whether their nutritional properties are retained, and whether the final products are safe to consume (6). Research on upcycled food acceptance has therefore examined factors such as environmental awareness, health consciousness, food novelty, perceived naturalness, perceived risk, disgust, product knowledge, and subjective norms (7–9). These studies provide important insights into why consumers may support or resist upcycled foods. Nevertheless, much of this literature has focused on relatively stable consumer characteristics or product attributes, while paying less attention to the digital communication environments in which consumers first encounter, interpret, and evaluate these unfamiliar products.

This issue has become increasingly relevant as short-video platforms, livestreaming commerce, and content-based e-commerce shape how food information is communicated. In this study, an AI virtual streamer refers to a digitally generated communicator that uses artificial intelligence technologies to present product information and interact with consumers in short-video or livestreaming environments (10, 11). Such streamers can introduce products, answer consumer questions, maintain continuous interaction, and deliver consistent brand messages (12). Existing research has examined a range of AI virtual streamer and virtual influencer characteristics, including expertise, interactivity, anthropomorphism, attractiveness, entertainment, authenticity, social presence, and personalization, as well as their effects on brand attitudes, engagement, trust, and purchase intention (12, 13). Yet most studies have focused on general consumer goods or broad livestreaming contexts. Less is known about how AI virtual streamers may help consumers evaluate sustainable food innovations that involve both cognitive unfamiliarity and heightened sensitivity to food safety.

The present study focuses on three characteristics that represent distinct communication cues. Expertise refers to the AI virtual streamer’s product knowledge and ability to provide clear and accurate explanations (14). In an upcycled food context, this may involve explaining that apple pomace used in a snack product comes from juice processing, describing how the material is screened and processed, or clarifying its nutritional and sustainability benefits. Interactivity refers to the streamer’s ability to respond to consumers’ questions and provide relevant information during communication (15). For example, consumers may ask whether an upcycled ingredient is food-grade, whether nutrients are retained after processing, or how the product differs from food made from expired or discarded materials. Anthropomorphism refers to the extent to which the AI virtual streamer uses human-like language, expressions, tone, and interaction styles (16). These features may make communication feel more natural and reduce the psychological distance associated with interacting with an artificial agent. Together, expertise, interactivity, and anthropomorphism capture the informational, interactional, and social dimensions of AI-mediated food communication. Other characteristics, such as visual attractiveness or entertainment, may be more closely associated with attention and hedonic engagement (17), whereas the present study focuses on how consumers understand and evaluate an unfamiliar and safety-sensitive food category.

The psychological mechanisms through which these characteristics operate also require closer examination. This study focuses on perceived value and product trust because they represent two different evaluations that are especially relevant to upcycled foods. Perceived value refers here to consumers’ overall assessment of whether the product provides worthwhile consumption, health and nutritional, functional, and environmental benefits (18). Product trust refers to consumers’ confidence that the product is safe, that its ingredient sources are credible, and that its processing and quality controls are reliable (4). Perceived value therefore captures benefit appraisal, whereas product trust captures uncertainty reduction. These mechanisms are related but not interchangeable. A consumer may recognize the environmental value of a snack made from fruit-processing by-products but remain reluctant to purchase it because of concerns about ingredient safety or processing (19). Conversely, a product may be considered safe and reliable but still be rejected if consumers see little nutritional, functional, or practical advantage over conventional alternatives (20).

Despite growing research on upcycled food acceptance and AI-enabled marketing communication (21), the correspondence between different AI virtual streamer characteristics and different psychological mechanisms remains insufficiently integrated. Expertise, interactivity, and anthropomorphism have often been examined separately, or their effects have been explained through a single mechanism such as trust, social presence, or perceived value (22, 23). It therefore remains unclear whether informational, interactional, and social cues perform different functions in shaping consumer evaluations. This gap is particularly relevant to upcycled foods because consumers must simultaneously determine whether a product is worth choosing and whether it can be trusted. Expertise and interactivity may provide diagnostic and responsive information that supports both benefit appraisal and uncertainty reduction, whereas anthropomorphism may function more strongly as a relational cue that reduces psychological distance and facilitates trust formation (12, 24, 25). The central research problem is therefore how distinct AI virtual streamer characteristics activate perceived value and product trust and whether these mechanisms form different pathways to purchase intention.

To address this problem, the present study draws on the stimulus–organism–response (SOR) framework and develops a model in which AI virtual streamer expertise, interactivity, and anthropomorphism represent external stimuli; perceived value and product trust represent consumers’ internal psychological responses; and purchase intention toward upcycled foods represents the behavioral response. A three-wave time-lagged survey design was employed to examine these relationships. Specifically, the study addresses the following research questions:

*RQ1*: How do AI virtual streamer expertise, interactivity, and anthropomorphism influence consumers’ perceived value and product trust in upcycled foods?

*RQ2*: Do perceived value and product trust provide distinct psychological pathways through which AI virtual streamer characteristics shape consumers’ purchase intention toward upcycled foods?

*RQ3*: Do the indirect effects of expertise, interactivity, and anthropomorphism differ across the perceived-value and product-trust pathways?

This study makes three main contributions. First, it extends research on upcycled food acceptance by shifting attention from consumer traits and product attributes to the AI-mediated communication environment in which consumers interpret these products. Second, it distinguishes expertise, interactivity, and anthropomorphism as informational, interactional, and social cues rather than treating AI virtual streamer characteristics as uniformly beneficial. Third, it differentiates benefit appraisal from uncertainty reduction within the SOR framework by examining perceived value and product trust as related but distinct psychological mechanisms. This approach provides a more precise account of how AI-enabled food communication may shape consumer acceptance of upcycled foods and offers practical guidance for product explanation, interaction design, and trust building in sustainable food marketing.

## 2 Literature review and hypothesis development

### 2.1 Consumer acceptance of upcycled foods

Consumer acceptance is essential to the market development of upcycled foods because the environmental benefits of food upcycling can only be realized when consumers are willing to incorporate these products into their food choices (26). Although upcycled foods contribute to food waste reduction and resource circulation, they remain unfamiliar to many consumers and may evoke competing evaluations (4). Consumers may associate them with sustainability, innovation, and responsible consumption, while also questioning whether ingredients derived from underused materials are safe, natural, nutritious, and of acceptable quality (27). These competing evaluations help explain why favorable attitudes toward food waste reduction do not necessarily translate into willingness to purchase upcycled foods.

Previous research has identified three broad groups of factors associated with consumer acceptance. The first concerns individual characteristics. Environmental awareness, food waste awareness, health consciousness, product knowledge, familiarity, and openness to food innovation are generally associated with more favorable responses, whereas food neophobia, food technology neophobia, risk aversion, and disgust may inhibit acceptance (26, 28–30). The second group concerns product characteristics, including ingredient origin, nutritional quality, taste, appearance, price, processing methods, and perceived naturalness (31, 32). Because upcycled foods may be made from food-processing by-products or other underused edible materials, consumers evaluate not only their environmental benefits but also whether they meet conventional expectations regarding safety, sensory quality, nutrition, and everyday consumption utility. Recent studies further indicate that health, economic, emotional, epistemic, and environmental values may contribute differently to attitudes and purchase intention (33, 34).

The third group concerns information and communication. Before purchase, consumers usually cannot directly verify how upcycled ingredients were sourced, screened, processed, or quality-controlled. They therefore rely partly on external cues such as ingredient descriptions, labels, certifications, nutritional information, environmental claims, and explanations of the upcycling process (35, 36). For example, information explaining that fruit pomace originates from juice production, remains suitable for food use after screening, and is subsequently processed into a new snack may help consumers distinguish food upcycling from the reuse of unsafe or expired materials. Likewise, information about nutrient retention, food-safety controls, and waste-reduction benefits may reduce uncertainty and strengthen product evaluation (9, 37). The effectiveness of such information, however, may depend on the concerns consumers bring to the evaluation. Environmental claims may be meaningful to sustainability-oriented consumers, whereas those primarily concerned about safety, taste, or processing may require more specific and verifiable information.

Existing research therefore provides substantial evidence regarding which consumers are more receptive to upcycled foods and which product and information attributes contribute to acceptance. However, most studies have focused on consumer traits, product characteristics, labels, message framing, or relatively static forms of information provision. Less attention has been paid to interactive digital environments in which product information can be explained dynamically and consumer questions can be addressed during the communication process. Short-video and livestreaming platforms create such conditions and increasingly use AI-based communicators to deliver product information. It remains insufficiently understood how the characteristics of these communicators shape consumers’ evaluations of upcycled foods, particularly their judgments of product value and trustworthiness.

### 2.2 AI virtual streamer characteristics

As introduced earlier, AI virtual streamers are digitally generated communicators that use artificial intelligence technologies to present product information and interact with audiences in livestreaming or short-video environments (10). Unlike static product pages, they combine visual presentation, verbal explanation, and interactive communication within a digital persona. They also differ from human streamers because their appearance, scripts, expressions, and response patterns can be designed and managed algorithmically (38). Research has increasingly moved beyond comparisons between human and virtual streamers to examine which characteristics of AI-mediated communicators shape consumer responses.

Existing studies have approached these characteristics from several perspectives. Information- and source-related characteristics include expertise, competence, credibility, and authenticity, which indicate whether the communicator can provide knowledgeable and credible product information (12, 39). Communication-related characteristics include interactivity, responsiveness, vividness, telepresence, and personalization (40, 41). In this study, interactivity is understood more narrowly as the extent to which an AI virtual streamer can acknowledge consumer questions and provide timely and relevant responses during communication. Social and representational characteristics include anthropomorphism, attractiveness, likeability, animacy, and social presence, which influence how human-like and socially present the virtual communicator appears (42, 43). Prior research suggests that interactive communication can influence social presence and perceived value (11), whereas anthropomorphic cues may shape credibility, psychological distance, parasocial relationships, and trust (44, 45).

The present study focuses on expertise, interactivity, and anthropomorphism because they capture three distinct functions that are directly relevant to upcycled food communication. Expertise represents the streamer’s ability to provide accurate and understandable product information; interactivity captures its ability to respond to specific consumer concerns; and anthropomorphism reflects the degree to which its language, expressions, tone, and interaction style resemble human communication (40, 46). These characteristics correspond to informational, interactional, and social cues, respectively. Their distinction is particularly relevant to upcycled foods, where consumers may need factual explanations of ingredient sources and processing, answers to questions about nutrition and safety, and a communication style that reduces the unfamiliarity associated with both the product and the AI communicator (38). Other characteristics, such as visual attractiveness or entertainment, may be more closely related to attention or enjoyment. The present study instead examines characteristics that are directly connected to product understanding, uncertainty reduction, and purchase-related evaluation. Although previous studies have linked AI virtual streamer characteristics to engagement and purchase intention (15, 47), how these different cues correspond to different psychological mechanisms in sustainable food contexts remains insufficiently integrated.

### 2.3 SOR framework

The SOR framework explains behavior as a response that develops through internal psychological processing after exposure to external stimuli (48). A stimulus refers to an external cue that influences an individual’s internal state, the organism represents the cognitive and affective processes through which that cue is interpreted, and the response refers to the resulting behavioral tendency. The framework has been widely applied in digital marketing, livestreaming commerce, and technology-mediated consumer research to explain how technological features, information cues, and interaction experiences shape internal evaluations such as perceived value, trust, social presence, and perceived risk, which subsequently influence behavioral intentions (49, 50).

In this study, AI virtual streamer expertise, interactivity, and anthropomorphism are treated as external stimuli. Expertise represents the informational cue provided by the streamer, particularly its ability to explain ingredient sources, processing methods, nutritional attributes, safety standards, and sustainability benefits (51). Interactivity refers specifically to the streamer’s ability to acknowledge consumer questions and provide timely and relevant responses during communication, rather than to interaction in a broad technological sense. Anthropomorphism represents the degree to which the streamer uses human-like language, expressions, tone, and communication styles, making the interaction appear more socially natural and approachable (52). Perceived value and product trust are positioned as the organism components because they represent two distinct internal evaluations involved in upcycled food acceptance (33, 34). Perceived value is defined here as consumers’ overall assessment of the consumption, health and nutritional, environmental, and general benefits of an upcycled food, whereas product trust refers to consumers’ confidence in its safety, ingredient credibility, processing standards, and overall reliability.

Purchase intention toward upcycled foods represents the response component of the model. The proposed SOR process suggests that consumers first encounter informational, interactional, and social cues from the AI virtual streamer, then evaluate whether the product is worthwhile and trustworthy, and finally form purchase intention. By incorporating perceived value and product trust simultaneously, this study distinguishes benefit appraisal from uncertainty reduction rather than treating the organism component as a single psychological state. This formulation provides a more precise basis for examining whether different AI virtual streamer characteristics operate through different psychological pathways in shaping consumer acceptance of upcycled foods.

### 2.4 Hypothesis development

#### 2.4.1 AI virtual streamer characteristics and perceived value

The consistent presentation and continuous communication of AI virtual streamers provide consumers with repeated opportunities to understand the benefits of unfamiliar food products. Expertise enables an AI virtual streamer to explain ingredient sources, processing methods, nutritional attributes, safety standards, and sustainability benefits (53), helping consumers recognize that upcycled foods are value-enhanced products rather than foods made from inferior or discarded ingredients. Interactivity allows the streamer to respond to consumers’ specific concerns about safety, nutrition, taste, processing, and food waste reduction (54), thereby transforming standardized product claims into information that is more relevant to individual needs. Anthropomorphism, reflected in human-like language, expressions, tone, and communication style, may make product information feel more natural and accessible, reduce psychological distance, and help consumers relate the benefits of upcycled foods to familiar consumption situations (55). Expertise therefore strengthens consumers’ understanding of product benefits, interactivity makes those benefits more personally relevant, and anthropomorphism facilitates the reception and processing of value-related information. Together, these characteristics may enhance consumers’ overall perceived value of upcycled foods. Accordingly, this study proposes:

*H1a*: AI virtual streamer expertise is positively associated with consumers’ perceived value of upcycled foods.

*H1b*: AI virtual streamer interactivity is positively associated with consumers’ perceived value of upcycled foods.

*H1c*: AI virtual streamer anthropomorphism is positively associated with consumers’ perceived value of upcycled foods.

#### 2.4.2 AI virtual streamer characteristics and product trust

Consumers often have limited knowledge of how upcycled foods are sourced, processed, and quality-controlled, making external communication cues important for trust formation. Expertise enables an AI virtual streamer to provide clear and credible explanations regarding ingredient origins, processing procedures, food safety, nutritional retention, and quality standards (56), thereby giving consumers a stronger basis for judging product reliability. Interactivity allows consumers to raise specific concerns and receive relevant responses, reducing uncertainty caused by incomplete or one-way information (57). Anthropomorphism, reflected in natural language, human-like expressions, tone, and communication style, may make the AI virtual streamer appear more approachable and socially present (58), thereby reducing psychological distance and increasing consumers’ willingness to accept product information. Expertise therefore strengthens the credibility of product explanations, interactivity reassures consumers that their concerns can be addressed, and anthropomorphism creates a more natural communication environment for trust formation. Together, these characteristics may enhance consumers’ trust in the upcycled foods recommended by AI virtual streamers. Accordingly, this study proposes:

*H2a*: AI virtual streamer expertise is positively associated with consumers’ product trust in upcycled foods.

*H2b*: AI virtual streamer interactivity is positively associated with consumers’ product trust in upcycled foods.

*H2c*: AI virtual streamer anthropomorphism is positively associated with consumers’ product trust in upcycled foods.

#### 2.4.3 Perceived value, product trust, and purchase intention

Consumers’ purchase intention toward upcycled foods depends on both whether they consider the product worthwhile and whether they believe it can be consumed with confidence. Perceived value reflects consumers’ overall evaluation of the product’s consumption, nutritional, functional, and environmental benefits (59). When these benefits are seen as relevant and sufficient, consumers are more likely to regard upcycled foods as meaningful alternatives to conventional products. Product trust addresses a different concern by reducing uncertainty about ingredient sources, processing procedures, quality control, and food safety (6). Even when consumers recognize the sustainability benefits of upcycled foods, doubts about reliability may prevent them from considering a purchase. Perceived value therefore strengthens consumers’ motivation by clarifying why the product is worth choosing, whereas product trust lowers the psychological barrier associated with whether the product is safe and dependable (60, 61). Together, these evaluations help transform favorable perceptions of upcycled foods into purchase intention. Accordingly, this study proposes:

*H3*: Consumers’ perceived value of upcycled foods is positively associated with their purchase intention.

*H4*: Consumers’ product trust in upcycled foods is positively associated with their purchase intention.

#### 2.4.4 The mediating roles of perceived value and product trust

Expertise can make nutritional, functional, and environmental benefits more understandable, interactivity can connect product information with consumers’ specific concerns, and anthropomorphism can make the communication process more natural and accessible (42, 46). These characteristics may therefore increase purchase intention by strengthening consumers’ overall assessment of whether the product is worth choosing. At the same time, expertise can provide credible information about ingredients and processing, interactivity can reduce uncertainty by addressing consumers’ questions, and anthropomorphism can lower psychological distance and create a more approachable communication environment (12, 62). These cues may consequently increase purchase intention by strengthening consumers’ confidence in product safety and reliability. Perceived value and product trust thus represent two related but distinct mechanisms: perceived value translates AI virtual streamer characteristics into stronger benefit appraisal (11), whereas product trust translates them into lower product-related uncertainty (63). Accordingly, this study proposes:

*H5a*: Perceived value mediates the relationship between AI virtual streamer expertise and consumers’ purchase intention toward upcycled foods.

*H5b*: Perceived value mediates the relationship between AI virtual streamer interactivity and consumers’ purchase intention toward upcycled foods.

*H5c*: Perceived value mediates the relationship between AI virtual streamer anthropomorphism and consumers’ purchase intention toward upcycled foods.

*H6a*: Product trust mediates the relationship between AI virtual streamer expertise and consumers’ purchase intention toward upcycled foods.

*H6b*: Product trust mediates the relationship between AI virtual streamer interactivity and consumers’ purchase intention toward upcycled foods.

*H6c*: Product trust mediates the relationship between AI virtual streamer anthropomorphism and consumers’ purchase intention toward upcycled foods.

**FIGURE 1:**
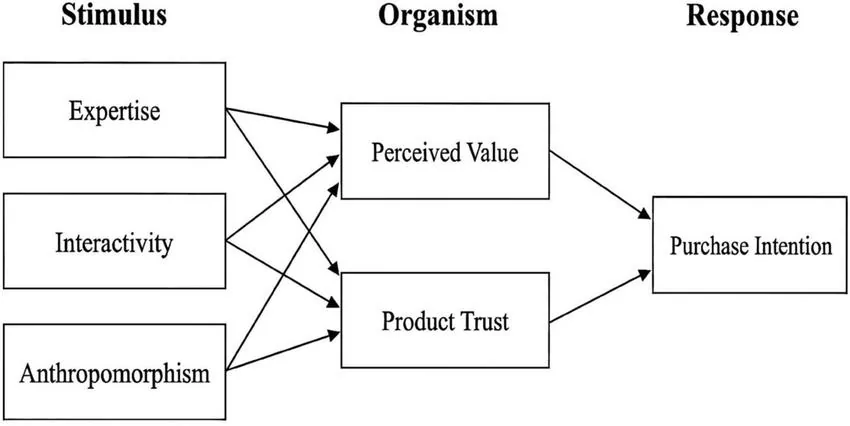
Research model.

On this basis, this study develops the research model shown in Figure 1.

## 3 Research design

### 3.1 Sample and procedure

The target population comprised adult consumers in China with experience using short-video, livestreaming e-commerce, or content-commerce platforms. This population was selected because respondents needed sufficient familiarity with digital product-recommendation settings to understand a scenario in which an AI virtual streamer recommends an upcycled food product. Eligible participants were required to be at least 18 years old, have experience using at least one of these platforms, and demonstrate an adequate understanding of the research scenario through the screening questions. A non-probability sampling approach combining convenience and snowball sampling was adopted.

The questionnaires were administered through Wenjuanxing,^1^ a widely used online survey platform in China. Potential respondents were recruited through social media and online consumer communities, and initial participants were encouraged to share the survey invitation with other eligible consumers. Before beginning each survey, respondents were informed that the study was conducted solely for academic purposes, participation was voluntary, responses would be processed anonymously, and they could withdraw at any time. Participants received RMB 2 for completing each survey wave. A brief explanation of upcycled foods and a scenario describing an AI virtual streamer recommending such a product were provided before the measurement items. Screening and attention-check questions were also included to identify ineligible or inattentive responses.

Data were collected in three waves separated by 1-week intervals. Wave 1 measured AI virtual streamer expertise, interactivity, anthropomorphism, and demographic characteristics. Wave 2 assessed perceived value and product trust among respondents who passed the Wave 1 screening, and Wave 3 measured purchase intention toward upcycled foods among respondents with valid Wave 2 responses. This temporal separation was consistent with the proposed SOR sequence and helped reduce potential common method bias associated with measuring all constructs at the same time. Responses across waves were matched using anonymous identification codes created by the respondents according to the same instructions in each survey. No personally identifiable information was collected. At each wave, responses were screened for eligibility, failed attention checks, implausibly short completion times, patterned responding, and inconsistent identification codes.

The sample flow is summarized in Table 1. In Wave 1, 547 questionnaires were returned, of which 482 remained after data screening and were invited to participate in Wave 2. A total of 466 Wave 2 questionnaires were returned, with 435 retained after quality checks. These 435 respondents were subsequently invited to Wave 3, yielding 421 returned questionnaires. After applying the same screening criteria and matching anonymous identification codes across all three waves, 392 respondents had complete and valid data at every measurement occasion. Thus, the final sample of 392 represents a matched three-wave sample rather than the combined number of responses collected across the three surveys. The final retention rate was 81.3% relative to the 482 valid respondents retained in Wave 1.

**TABLE 1:** Sample flow across the three survey waves.

Survey stage	Respondents invited	Questionnaires returned	Valid responses
Wave 1	–	547	482
Wave 2	482	466	435
Wave 3	435	421	392
Final matched sample	–	–	392

Among the 392 respondents in the final matched sample, 185 were male (47.2%) and 207 were female (52.8%). Regarding age, 79 respondents were aged 18–25 years (20.2%), 101 were aged 26–35 years (25.8%), 93 were aged 36–45 years (23.7%), 67 were aged 46–55 years (17.1%), and 52 were older than 55 years (13.3%). In terms of education, 77 respondents had completed high school or below (19.6%), 108 had a junior college education (27.6%), 115 held an undergraduate degree (29.3%), and 92 held a master’s degree or above (23.5%). Monthly income was below RMB 5,000 for 96 respondents (24.5%), RMB 5,000–10,000 for 119 respondents (30.4%), RMB 10,001–15,000 for 94 respondents (24.0%), and above RMB 15,000 for 83 respondents (21.2%). Gender, age, education, and monthly income were subsequently included as control variables in the structural model.

### 3.2 Measurement

The measurement items were adapted from previously published scales and modified to fit the context of AI virtual streamers recommending upcycled foods. All items were assessed using a five-point Likert scale ranging from 1 (“strongly disagree”) to 5 (“strongly agree”). The wording was adjusted to refer explicitly to the AI virtual streamer and the upcycled food being recommended while preserving the conceptual meaning of the original measures.

AI virtual streamer expertise, interactivity, and anthropomorphism were measured using items adapted from Li and Huang (64), Tian et al. (65), and Yu et al. (66). Expertise was assessed with four items capturing the streamer’s ability to explain ingredient sources and product characteristics, provide professional information about safety, nutrition, and processing, demonstrate knowledge of upcycled foods, and facilitate consumer understanding of the product. Interactivity was measured with four items assessing whether the streamer could provide targeted and timely responses to consumer questions, involve consumers in the communication process, and provide additional information in response to specific concerns. Anthropomorphism was assessed with four items capturing the extent to which the streamer’s language, expressions, tone, movements, and communication style were perceived as natural, human-like, friendly, and similar to interaction with a human recommender.

Perceived value was measured using four items adapted from Nguyen et al. (67) and Slack et al. (68). The items assessed perceived consumption value, contribution to reducing food and resource waste, health/nutritional/functional value, and the overall value of choosing the upcycled food. Product trust was measured using four items adapted from Wu and Huang (61) and Jiang et al. (69). The items assessed confidence in product safety and reliability, ingredient sources and processing, product quality, and overall trust in the recommended upcycled food. Purchase intention was measured using four items adapted from Li and Shan (70) and Watanabe et al. (71), covering willingness to purchase, priority consideration, intention to try the product in the future, and willingness to recommend it to others.

The final measurement instrument therefore contained six latent constructs and 24 items. The complete wording of all items is presented in Table 2.

**TABLE 2:** Measurement items.

Construct	Code	Item
Expertise	EXP1	The AI virtual streamer can clearly explain the ingredient sources and product characteristics of the upcycled food it recommends.
	EXP2	The AI virtual streamer can provide professional information about the safety, nutrition, and processing of the upcycled food it recommends.
	EXP3	The AI virtual streamer demonstrates a high level of expertise in upcycled foods.
	EXP4	The AI virtual streamer’s introduction helps me better understand the upcycled food it recommends.
Interactivity	INT1	The AI virtual streamer can provide targeted responses to consumers’ questions about the upcycled food it recommends.
	INT2	The AI virtual streamer can promptly respond to consumers’ questions about the upcycled food it recommends.
	INT3	The AI virtual streamer’s interactive style makes me feel involved in the process of learning about upcycled foods.
	INT4	The AI virtual streamer can provide additional product information based on consumers’ concerns.
Anthropomorphism	ANT1	The AI virtual streamer’s language expression is natural and similar to human communication.
	ANT2	The AI virtual streamer’s expressions, tone, or movements make it seem human-like.
	ANT3	When watching the AI virtual streamer introduce upcycled foods, I feel as if I am communicating with a real recommender.
	ANT4	The AI virtual streamer’s way of expression feels friendly and approachable.
Perceived value	PV1	I think the upcycled food recommended by the AI virtual streamer has high consumption value.
	PV2	I think purchasing the upcycled food recommended by the AI virtual streamer can help reduce food waste and resource waste.
	PV3	I think the upcycled food recommended by the AI virtual streamer has certain value in terms of health, nutrition, or functionality.
	PV4	Overall, I think the upcycled food recommended by the AI virtual streamer is a valuable consumption choice.
Product trust	PT1	I believe that the upcycled food recommended by the AI virtual streamer is safe and reliable.
	PT2	I believe that the ingredient sources and processing of the upcycled food recommended by the AI virtual streamer are trustworthy.
	PT3	I believe that the upcycled food recommended by the AI virtual streamer has stable and acceptable product quality.
	PT4	Overall, I am willing to trust the upcycled food recommended by the AI virtual streamer.
Purchase intention	PI1	I would be willing to purchase the upcycled food recommended by the AI virtual streamer.
	PI2	I would give priority to the upcycled food recommended by the AI virtual streamer.
	PI3	I plan to try purchasing the upcycled food recommended by the AI virtual streamer in the future.
	PI4	I would be willing to recommend the upcycled food recommended by the AI virtual streamer to others.

## 4 Results

### 4.1 Common method bias test

For the statistical assessment of common method bias, this study first conducted Harman’s single-factor test. The results showed that the first unrotated factor explained 42.215% of the total variance, which was below the commonly used threshold of 50% (72). This suggests that common method bias was not a serious concern in the data. Because Harman’s single-factor test provides only a preliminary and relatively coarse assessment, this study further compared the fit of a single-factor CFA model with that of the proposed multi-factor CFA model. The single-factor CFA model showed poor fit: χ^2^/df = 10.222, RMSEA = 0.154, SRMR = 0.110, CFI = 0.624, and TLI = 0.588. By contrast, the proposed multi-factor model demonstrated good fit: χ^2^/df = 1.993, RMSEA = 0.050, SRMR = 0.035, CFI = 0.962, and TLI = 0.956. The clear difference between the two models indicates that the variables could not be adequately explained by a single common factor.

Taken together, the Harman’s single-factor test result was below the 50% reference criterion, the single-factor CFA model fitted the data poorly, and the proposed multi-factor model showed acceptable fit. These findings indicate that common method bias did not seriously undermine the distinctiveness of the constructs in this study. Considering the procedural controls adopted in the questionnaire design, including temporal separation, anonymous responses, and data quality checks, common method bias was considered to be within an acceptable range.

### 4.2 Collinearity diagnosis

This study used the variance inflation factor (VIF) and tolerance values to assess collinearity among the measurement items (73). As shown in Table 3, the VIF values ranged from 1.920 to 4.490, all below the commonly used threshold of 5. The tolerance values ranged from 0.223 to 0.521, all above 0.10. Overall, the results suggest that there was no serious collinearity problem in the data. Although some correlations existed among the items, the measurement items still retained sufficient statistical distinctiveness for subsequent analyses.

**TABLE 3:** Collinearity diagnosis.

Item	VIF	Tolerance
EXP1	2.093	0.478
EXP2	2.042	0.490
EXP3	1.920	0.521
EXP4	2.051	0.488
INT1	2.076	0.482
INT2	2.006	0.498
INT3	1.933	0.517
INT4	2.183	0.458
ANT1	2.174	0.460
ANT2	2.108	0.474
ANT3	2.096	0.477
ANT4	2.182	0.458
PV1	3.904	0.256
PV2	3.459	0.289
PV3	3.748	0.267
PV4	3.620	0.276
PT1	3.669	0.273
PT2	4.490	0.223
PT3	4.468	0.224
PT4	3.518	0.284
PI1	2.950	0.339
PI2	2.824	0.354
PI3	2.325	0.430
PI4	2.350	0.426

EXP, expertise; INT, interactivity; ANT, anthropomorphism; PV, perceived value; PT, product trust; PI, purchase intention.

### 4.3 Reliability and validity tests

The reliability and convergent validity of the measurement model were assessed using standardized factor loadings, Cronbach’s α, composite reliability (CR), and average variance extracted (AVE). Cronbach’s α and CR values of 0.70 or higher are generally considered to indicate satisfactory internal consistency, while an AVE value of at least 0.50 indicates that a construct explains at least half of the variance in its indicators (74, 75). Standardized factor loadings around 0.70 or higher are also generally regarded as evidence that the indicators adequately represent their intended latent constructs (75).

As shown in Table 4, Cronbach’s α values ranged from 0.824 to 0.927 and CR values ranged from 0.825 to 0.927, with all values exceeding the recommended threshold of 0.70. The standardized factor loadings ranged from 0.700 to 0.896, indicating adequate indicator reliability. AVE values ranged from 0.541 to 0.760, all exceeding the recommended minimum of 0.50. Taken together, these results indicate satisfactory internal consistency and convergent validity across all six constructs.

**TABLE 4:** Factor loading, Cronbach’s α, AVE, and CR.

Construct	Item	Factor loading	Cronbach’s α	AVE	CR
Expertise	EXP1	0.767	0.834	0.557	0.834
	EXP2	0.740			
	EXP3	0.735			
	EXP4	0.744			
Interactivity	INT1	0.736	0.824	0.541	0.825
	INT2	0.727			
	INT3	0.700			
	INT4	0.778			
Anthropomorphism	ANT1	0.762	0.839	0.566	0.839
	ANT2	0.748			
	ANT3	0.751			
	ANT4	0.749			
Perceived value	PV1	0.880	0.926	0.759	0.926
	PV2	0.860			
	PV3	0.876			
	PV4	0.868			
Product trust	PT1	0.855	0.927	0.760	0.927
	PT2	0.896			
	PT3	0.891			
	PT4	0.846			
Purchase intention	PI1	0.859	0.891	0.674	0.892
	PI2	0.848			
	PI3	0.778			
	PI4	0.795			

AVE, average variance extracted; CR, composite reliability.

Discriminant validity was assessed using the Fornell–Larcker criterion (74). According to this criterion, discriminant validity is supported when the square root of the AVE for each construct is greater than its correlations with all other constructs. As shown in Table 5, the square roots of the AVE ranged from 0.736 to 0.872 and exceeded all corresponding inter-construct correlations. For example, the square root of the AVE for perceived value was 0.871, which was greater than its highest correlation with another construct, product trust (*r* = 0.679). Similarly, the square root of the AVE for product trust was 0.872, exceeding its correlations with all other constructs. These results provide evidence of satisfactory discriminant validity.

**TABLE 5:** Pearson correlations and square roots of AVE.

Construct	EXP	INT	ANT	PV	PT	PI
EXP	0.747	0.736	0.752	0.871	0.872	0.821
INT	0.586					
ANT	0.563	0.582				
PV	0.479	0.472	0.402			
PT	0.557	0.525	0.545	0.679		
PI	0.328	0.349	0.297	0.444	0.449	

Diagonal values in bold are the square roots of AVE. Off-diagonal values are Pearson correlation coefficients. EXP, expertise; INT, interactivity; ANT, anthropomorphism; PV, perceived value; PT, product trust; PI, purchase intention.

### 4.4 Structural equation modeling analysis

This study used structural equation modeling to test the proposed hypotheses. Gender, age, education level, and monthly income were included as control variables to reduce the potential influence of demographic differences on path estimates. The structural model showed acceptable fit: χ^2^/df = 2.403, RMSEA = 0.060, SRMR = 0.065, CFI = 0.945, and TLI = 0.937. These results indicate that the theoretical model fitted the sample data adequately, allowing further analysis of the hypothesized paths among the variables (76).

As shown in Table 6, AI virtual streamer expertise had a significant positive effect on perceived value (β = 0.357, *p* < 0.001), supporting H1a. AI virtual streamer interactivity also had a significant positive effect on perceived value (β = 0.286, *p* = 0.001), supporting H1b. These results indicate that both expertise and interactivity significantly predicted consumers’ perceived value of upcycled foods. By contrast, the effect of AI virtual streamer anthropomorphism on perceived value was not significant (β = 0.046, *p* = 0.572), and H1c was not supported. This suggests that perceived value was not equally influenced by all AI virtual streamer characteristics, with expertise and interactivity showing more evident effects.

**TABLE 6:** Model regression coefficients.

Hypothesis	Path	SE	*z*	*p*	β
H1a	EXP → PV	0.112	4.239	0.000	0.357
H1b	INT → PV	0.120	3.270	0.001	0.286
H1c	ANT → PV	0.107	0.565	0.572	0.046
H2a	EXP → PT	0.098	4.643	0.000	0.360
H2b	INT → PT	0.104	2.615	0.009	0.208
H2c	ANT → PT	0.094	3.134	0.002	0.233
H3	PV → PI	0.057	5.126	0.000	0.292
H4	PT → PI	0.060	5.297	0.000	0.302

EXP, expertise; INT, interactivity; ANT, anthropomorphism; PV, perceived value; PT, product trust; PI, purchase intention.

For product trust, AI virtual streamer expertise had a significant positive effect (β = 0.360, *p* < 0.001), supporting H2a. Interactivity also had a significant positive effect on product trust (β = 0.208, *p* = 0.009), supporting H2b. Anthropomorphism had a significant positive effect on product trust as well (β = 0.233, *p* = 0.002), supporting H2c. These findings indicate that consumers’ trust in upcycled foods was associated not only with information explanation and interactive responses, but also with the natural expression and human-like communication style of AI virtual streamers.

Furthermore, perceived value had a significant positive effect on purchase intention (β = 0.292, *p* < 0.001), supporting H3. Product trust also had a significant positive effect on purchase intention (β = 0.302, *p* < 0.001), supporting H4. These results suggest that both perceived value and product trust significantly predicted consumers’ purchase intention toward upcycled foods.

### 4.5 Mediation effect test

Building on the structural model results, this study further used the bootstrap method to examine the mediating roles of perceived value and product trust. An indirect effect was considered significant when the 95% bootstrap confidence interval did not include zero (77).

As shown in Table 7, for the perceived value pathway, the indirect effect of AI virtual streamer expertise on purchase intention through perceived value was 0.081, with a 95% BootCI of [0.033, 0.148]. Because the confidence interval did not include zero, H5a was supported. The indirect effect of AI virtual streamer interactivity on purchase intention through perceived value was 0.078, with a 95% BootCI of [0.031, 0.150], also supporting H5b. By contrast, the indirect effect of AI virtual streamer anthropomorphism on purchase intention through perceived value was 0.031, with a 95% BootCI of [-0.000, 0.089]. Since the confidence interval included zero, H5c was not supported. This result is consistent with the structural model results, in which anthropomorphism did not significantly predict perceived value and therefore did not form a stable indirect pathway through perceived value.

**TABLE 7:** Mediation effect test results.

Path	Total effect	Indirect effect	Boot SE	95% BootCI	Direct effect
EXP → PV→ PI	0.204*	0.081	0.029	0.033 ∼ 0.148	0.043
INT → PV → PI	0.276**	0.078	0.029	0.031 ∼ 0.150	0.139
ANT → PV → PI	0.116	0.031	0.022	-0.000 ∼ 0.089	0.010
EXP → PT→ PI	0.204*	0.081	0.033	0.025 ∼ 0.156	0.043
INT → PT → PI	0.276**	0.058	0.026	0.019 ∼ 0.126	0.139
ANT → PT → PI	0.116	0.076	0.031	0.026 ∼ 0.153	0.010

EXP, expertise; INT, interactivity; ANT, anthropomorphism; PV, perceived value; PT, product trust; PI, purchase intention; *p < 0.05, **p < 0.01.

For the product trust pathway, the indirect effect of AI virtual streamer expertise on purchase intention through product trust was 0.081, with a 95% BootCI of [0.025, 0.156], supporting H6a. The indirect effect of AI virtual streamer interactivity on purchase intention through product trust was 0.058, with a 95% BootCI of [0.019, 0.126], supporting H6b. The indirect effect of AI virtual streamer anthropomorphism on purchase intention through product trust was 0.076, with a 95% BootCI of [0.026, 0.153], supporting H6c. These findings indicate that product trust mediated the relationships between all three AI virtual streamer characteristics and purchase intention, suggesting that the product trust pathway was more complete in the proposed model.

## 5 Discussion

### 5.1 Research findings

Drawing on the SOR framework, this study examined how AI virtual streamer expertise, interactivity, and anthropomorphism shape consumers’ purchase intention toward upcycled foods through perceived value and product trust. The findings support the general SOR process in which external communication cues influence internal product evaluations, which subsequently predict behavioral intention. More importantly, the results do not indicate that all AI virtual streamer characteristics operate in the same way. Expertise and interactivity were positively associated with both perceived value and product trust, whereas anthropomorphism was associated only with product trust. This pattern suggests that informational, communicative, and social cues perform different functions when consumers evaluate an unfamiliar and safety-sensitive food category.

AI virtual streamer expertise was positively associated with perceived value (β = 0.357, *p* < 0.001) and product trust (β = 0.360, *p* < 0.001). These findings indicate that professional explanations are particularly important in the upcycled food context. Consumers may find it difficult to evaluate products made from underutilized ingredients because the sources of these ingredients, the upcycling process, and the resulting nutritional and environmental benefits are not immediately observable (31). When an AI virtual streamer can clearly explain ingredient origins, processing procedures, safety standards, nutritional attributes, and food-waste reduction benefits, consumers gain a stronger basis for judging both why the product is worthwhile and whether it is reliable (78). Expertise therefore functions as a product-diagnostic cue that supports benefit appraisal while reducing uncertainty about product quality and safety.

Interactivity was also positively associated with perceived value (β = 0.286, *p* = 0.001) and product trust (β = 0.208, *p* = 0.009). This result extends previous research on interactive digital communication by showing that responsiveness is relevant not only to engagement but also to consumers’ substantive evaluation of upcycled foods. Consumers may hold different concerns about ingredient safety, excessive processing, nutritional retention, taste, price, and the credibility of sustainability claims (79). One-way communication may leave these concerns unresolved, whereas interactive responses allow product information to be adjusted to consumers’ specific questions. Interactivity can therefore make product benefits more personally relevant and reduce the uncertainty created by incomplete information (80). In this sense, its value lies not simply in creating a more active communication experience, but in helping consumers convert general product claims into information they can assess.

The effect of anthropomorphism showed a different pattern. Anthropomorphism was positively associated with product trust (β = 0.233, *p* = 0.002), but its relationship with perceived value was not significant (β = 0.046, *p* = 0.572). This non-significant result is theoretically informative because it reveals a distinction between relational cues and product-diagnostic cues. Human-like language, expressions, tone, and communication style may make an AI virtual streamer appear more approachable and socially present, thereby reducing psychological distance and facilitating trust formation (16). However, these characteristics do not necessarily provide additional evidence regarding the nutritional, functional, environmental, or practical benefits of the product. Perceived value depends more strongly on information that helps consumers evaluate what the product offers (61), whereas anthropomorphism mainly changes how the communicator is socially perceived. A further explanation lies in the absence of embodied consumption experience. Even when an AI virtual streamer behaves in a highly human-like manner, consumers know that it cannot personally taste or consume the recommended food. Anthropomorphic presentation may therefore increase social closeness without strengthening the credibility of claims concerning taste, nutrition, or everyday consumption value (81).

Both perceived value (β = 0.292, *p* < 0.001) and product trust (β = 0.302, *p* < 0.001) positively predicted purchase intention. These results support the distinction between benefit appraisal and uncertainty reduction. Perceived value clarifies why upcycled foods are worth choosing by integrating consumption, nutritional, functional, and environmental benefits (82). Product trust lowers the psychological barrier created by concerns about ingredient origins, processing methods, quality control, and food safety (83). Consumers may recognize the sustainability benefits of an upcycled food but remain unwilling to purchase it if they do not trust the product. Conversely, they may regard the product as safe but still show limited purchase intention if its value is unclear. Purchase intention therefore depends on both recognizing sufficient benefits and developing confidence in product reliability.

The mediation results further clarify these two mechanisms. Perceived value mediated the relationships of expertise and interactivity with purchase intention, with indirect effects of 0.081 and 0.078, respectively. However, the indirect effect of anthropomorphism through perceived value was not significant. Product trust mediated the relationships of expertise, interactivity, and anthropomorphism with purchase intention, with indirect effects of 0.081, 0.058, and 0.076, respectively. These findings show that expertise and interactivity operate through both value recognition and trust formation, whereas anthropomorphism primarily operates through trust formation. The contribution of the study therefore lies not simply in confirming that perceived value and product trust predict purchase intention, but in demonstrating that different AI virtual streamer characteristics activate these psychological pathways unevenly. In the promotion of upcycled foods, informational and interactive cues support both product understanding and uncertainty reduction, while human-like social cues are more effective in creating trust than in demonstrating product value (84, 85).

### 5.2 Research contributions

The findings of this study contribute to research on AI-mediated communication, upcycled food consumption, and consumer decision-making in several ways. First, the study extends the empirical scope of upcycled food acceptance research by examining AI virtual streamers as a digital communication context. Previous studies have primarily explained consumer acceptance through individual characteristics, product attributes, perceived risk, environmental awareness, health consciousness, and food-related attitudes. These perspectives help explain why consumers may be receptive or resistant to upcycled foods, but they provide less insight into how consumers evaluate such products when information is delivered through emerging AI-mediated commerce environments. By examining expertise, interactivity, and anthropomorphism, this study shows that consumer responses are also associated with how unfamiliar food products are explained, communicated, and socially presented. The findings therefore broaden the study of upcycled food acceptance from relatively static consumer and product factors to the characteristics of the digital communicator involved in product evaluation.

Second, the results provide empirical support for distinguishing two internal evaluation processes within the SOR framework. Perceived value captures consumers’ assessment of whether an upcycled food offers worthwhile consumption, health and nutritional, functional, and environmental benefits, whereas product trust captures confidence in its safety, ingredient sources, processing standards, and overall reliability. Both were positively associated with purchase intention, but the three AI virtual streamer characteristics did not influence them in identical ways. The findings therefore suggest that benefit appraisal and uncertainty reduction should not be treated as interchangeable responses when examining AI-mediated communication of unfamiliar food products.

Third, the study identifies different empirical roles for expertise, interactivity, and anthropomorphism. Expertise and interactivity were positively associated with both perceived value and product trust, indicating that knowledgeable explanations and responsive communication may simultaneously help consumers recognize product benefits and reduce uncertainty. Anthropomorphism, however, was positively associated with product trust but not with perceived value. This pattern suggests that different AI communication cues may correspond to different consumer evaluations. Informational and interactional cues appear particularly relevant when consumers need product-specific information, whereas anthropomorphic cues may be more closely related to the relational and social aspects of trust formation.

The non-significant relationship between anthropomorphism and perceived value further qualifies the assumption that more human-like AI communication necessarily produces uniformly favorable consumer evaluations. In the context of upcycled foods, human-like language, expressions, and interaction styles may make an AI virtual streamer appear more approachable and trustworthy, but these features do not themselves provide evidence about nutritional quality, functional benefits, environmental performance, or other product attributes. Consumers may also recognize that an AI virtual streamer cannot personally taste or physically experience the food it recommends. The findings therefore indicate that the usefulness of anthropomorphic design may depend on the type of consumer response being considered. This distinction provides a more specific empirical basis for future research on AI-mediated communication in food and other products for which safety, quality, and product evaluation are particularly important.

### 5.3 Practical implications

The findings of this study provide several practical implications for upcycled food producers, retailers, AI virtual streamer designers, digital platforms, and food-sector regulators. First, firms should use AI virtual streamers to explain the upcycling process rather than relying primarily on general claims about sustainability or technological novelty. Consumers may confuse upcycled foods with expired products, low-quality leftovers, or foods produced from unsafe waste materials. AI virtual streamers should therefore provide clear information about where the ingredients originate, why they remain suitable for consumption, how they are screened and processed, whether their nutritional properties are retained, and how the final product contributes to food waste reduction. Visual demonstrations of ingredient transformation, production procedures, and quality testing may help consumers understand that upcycled foods are value-enhanced products rather than simple repackaging of discarded materials. In the Chinese digital commerce context, such explanations can be integrated into short videos, livestream demonstrations, product-detail pages, and traceability interfaces.

Second, interaction strategies should be designed around the concerns that consumers actually have about upcycled foods. The positive effects of interactivity on perceived value and product trust indicate that AI virtual streamers should not merely repeat standardized promotional messages. Firms can develop question-and-answer modules addressing issues such as whether the ingredients are food-grade, whether the product is excessively processed, how its nutrition and taste compare with conventional foods, why its price may differ, and how much food waste is reduced through production. By linking responses to specific product and production data, AI virtual streamers can convert vague concerns into information that consumers can evaluate. Interactive communication should therefore be treated as a mechanism for product clarification and uncertainty reduction rather than simply as a way to increase livestream engagement.

Third, firms should adopt a balanced approach to anthropomorphic design. Human-like language, facial expressions, tone, and communication style can make AI virtual streamers more approachable and may strengthen product trust. However, the non-significant effect of anthropomorphism on perceived value shows that human-like presentation cannot substitute for substantive product information. A highly anthropomorphic streamer may attract attention and make consumers more willing to engage with the recommendation, but consumers still need verifiable evidence concerning ingredients, nutrition, processing, quality, and environmental benefits. Anthropomorphic design should therefore support the delivery of credible information rather than become the main source of persuasion.

Fourth, trust building should extend beyond the AI virtual streamer itself. Because product trust mediated the effects of all three streamer characteristics on purchase intention, firms should provide information that consumers can independently verify. This may include ingredient-source records, food-safety standards, production and processing details, nutritional labels, third-party testing reports, certifications, and traceability QR codes. Claims about food waste reduction and environmental benefits should also be specific and measurable rather than vague or exaggerated. In China, where consumers are familiar with scanning QR codes and obtaining product information through mobile platforms, combining AI virtual streamer explanations with accessible traceability and testing information may be particularly effective in reducing concerns about product authenticity and safety.

Finally, digital platforms and regulators can support the development of the upcycled food market by improving information standards and consumer education. Platforms can require sellers to disclose standardized information on ingredient origins, processing methods, food-safety compliance, nutritional characteristics, and sustainability claims. They can also establish clear rules for AI identity disclosure and prevent virtual streamers from making unsupported health, safety, or environmental claims. Regulators and industry organizations may further develop labeling guidance, certification standards, and public education materials that distinguish upcycled foods from expired, defective, or low-quality products. These measures would strengthen the credibility of AI-mediated food communication and create a more reliable environment for consumer adoption of upcycled foods.

### 5.4 Limitations and future research

This study has several limitations that provide directions for future research. First, perceived value was operationalized as an overall evaluation that integrated consumption, health and nutritional, environmental, and general product benefits. This approach was suitable for examining perceived value as a parsimonious organism-level response within the SOR framework, but it may conceal differences among specific value dimensions. For example, expertise may be more strongly related to functional or nutritional value, whereas anthropomorphism may be more relevant to emotional or social value. Future research should adopt multiple items for each value dimension and examine multidimensional or second-order perceived value structures. Such an approach may provide a more precise explanation of how different AI virtual streamer characteristics shape different types of value perception.

Second, the non-significant relationship between anthropomorphism and perceived value suggests that the role of human-like AI design requires further investigation. The present study indicates that anthropomorphism may support trust formation without necessarily providing the diagnostic information needed for product-value evaluation. Future studies could examine whether this relationship changes when anthropomorphic presentation is combined with highly detailed product information, stronger perceived authenticity, or greater social presence. Researchers could also compare AI virtual streamers with human streamers to determine whether the absence of embodied food experience limits the persuasiveness of AI recommendations concerning taste, nutrition, and practical consumption value.

Finally, this study estimated the average structural relationships for the overall sample and did not test demographic heterogeneity. Age, education, gender, and income were included as control variables rather than theorized as moderators. It therefore remains unclear whether consumers with different demographic characteristics respond differently to AI virtual streamer expertise, interactivity, and anthropomorphism. Future research could use stratified sampling, larger subgroup samples, preregistered moderation hypotheses, and measurement invariance testing to examine potential differences across consumer groups. This would help identify whether particular AI communication strategies are more effective for specific market segments.

## Data Availability

The original contributions presented in the study are included in the article/supplementary material, further inquiries can be directed to the corresponding authors.

## Footnotes

1
www.wjx.cn

## References

[1] Boudalia S Symeon GK Dotas V Gueboudji Z Kouadri I Sehili B et al. The valorization of agrifood byproducts and waste to advance the sustainable development goals: current state and new perspectives. *Sustainability*. (2026) 18:2165. 10.3390/su18052165

[2] Hossain MS Wazed MA Preya MSA Sultana Z Kamal MM Ahmad T et al. Comprehensive review of biotechnological innovations in valorization of food waste: enhancing nutritional. *Food Front*. (2026) 7:e70194. 10.1002/fft2.70194

[3] Aschemann-Witzel J Asioli D Banovic M Perito MA Peschel AO Stancu V. Defining upcycled food: the dual role of upcycling in reducing food loss and waste. *Trends Food Sci Technol.* (2023) 132:132–7. 10.1016/j.tifs.2023.01.001

[4] Isaac-Bamgboye, Onyeaka H Isaac-Bamgboye IT Chukwugozie DC Afolayan M. Upcycling technologies for food waste management: safety, limitations, and current trends. *Green Chem Lett Rev.* (2025) 18:2533894. 10.1080/17518253.2025.2533894

[5] Coderoni S Perito MA. Sustainable consumption in the circular economy. an analysis of consumers’ purchase intentions for waste-to-value food. *J Clean Prod.* (2020) 252:119870. 10.1016/j.jclepro.2019.119870

[6] Meijer GW Lähteenmäki L Stadler RH Weiss J. Issues surrounding consumer trust and acceptance of existing and emerging food processing technologies. *Crit Rev Food Sci Nutr*. (2021) 61:97–115. 10.1080/10408398.2020.1718597 32003225

[7] Shan L Zhang Y Ma Y Tian J Li Y. Understanding purchase intention toward upcycled foods through a consumer education lens: an extended theory of planned behavior approach. *Front Nutr*. (2026) 13:1871592. 10.3389/fnut.2026.1871592 42568376 PMC13447081

[8] Šostar M Joy J Ramanathan H. Consumer trust in emerging food technologies: a comparative analysis of croatia and India. *Sustainability*. (2025) 17:7993. 10.3390/su17177993

[9] Zaman QU Rossetto L Cei L. Upcycled Foods: what influences consumer responses to a circular economy-based consumption strategy? *Foods.* (2026) 15:364. 10.3390/foods15020364 41596962 PMC12841369

[10] Cao Y Wang F. Generative AI streamers in action: a source credibility perspective. *Int J Advert.* (2025) 1–34. 10.1080/02650487.2025.2598980

[11] Liu H Zhang P Cheng H Hasan N Chiong R. Impact of AI-generated virtual streamer interaction on consumer purchase intention: a focus on social presence and perceived value. *J Retail Con Ser*. (2025) 85:104290. 10.1016/j.jretconser.2025.104290

[12] Wang L Yeap JAL Liu J Li Z. From avatars to algorithms: virtual streamers and ai-enabled consumer behavior in live streaming commerce—a systematic review. *J Theor App Electron Comm Res*. (2026) 21:57. 10.3390/jtaer21020057

[13] Tian M Hu H Chen M. Virtual influencers in interactive marketing: a state-of-art review and future research directions. *JRes Int Mark*. (2026) 20:794–811. 10.1108/jrim-03-2025-0123

[14] McCarthy DI. Nutritional intelligence in the food system: combining food, health, data and AI expertise. *Nutr Bull*. (2025) 50:142–50. 10.1111/nbu.12729 39799464 PMC11815607

[15] Han B Liu D Qi S. Beyond human streamers: how AI-avatar engagement strategies influence consumer acceptance. *Asia Pac J Mark Logist.* (2026) 38:2015–34. 10.1108/apjml-04-2025-0618

[16] Huo J Wang R Yang Q. When “nonsense” makes sense: effects of non-informational speech by virtual streamers in livestream commerce. *J Retailing Con Sers*. (2026) 92:104848. 10.1016/j.jretconser.2026.104848

[17] Li L Zhou W Chen Y. Factors influencing users’ watching intention in virtual streaming: the perspective of flow experience. *Kybernetes*. (2026) 55:895–921. 10.1108/k-03-2024-0721

[18] Thorsen M Skeaff S Goodman-Smith F Thong B Bremer P Mirosa M. Upcycled foods: a nudge toward nutrition. *Front Nutr*. (2022) 9:1071829. 10.3389/fnut.2022.1071829 36479294 PMC9719940

[19] Hasan MM Islam MR Haque AR Kabir MR Khushe KJ Hasan SMK. Trends and challenges of fruit by-products utilization: insights into safety, sensory, and benefits of the use for the development of innovative healthy food: a review. *Bioresour Bioprocess*. (2024) 11:10. 10.1186/s40643-023-00722-8 38647952 PMC10991904

[20] Moshtaghian H Bolton K Rousta K. Challenges for upcycled *foods*: definition. inclusion in the food waste management hierarchy and public acceptability. *Foods.* (2021) 10:2874. 10.3390/foods10112874 34829155 PMC8621107

[21] Parra-López C Carmona-Torres C. Enabling the circular food economy through industry 4.0: a review of applications. *Compr Rev Food Sci Food Saf*. (2026) 25:e70431. 10.1111/1541-4337.70431 41696763

[22] Shui X Bian S Zhang P. How can ai virtual streamers gain consumer trust to influence purchase intention in live-streaming e-commerce? *J Theor Appl Electron Comm Res*. (2025) 20:325. 10.3390/jtaer20040325

[23] Yu T Zeng Z Liu T Wang C. Virtual streamers, real purchases: the roles of anthropomorphism, interactivity, and presence in e-commerce live streaming. *Int J Contemp Hosp Manag*. (2026) 38:239–63. 10.1108/ijchm-02-2026-0155

[24] Chen M. Antecedents and outcomes of virtual presence in online shopping: A perspective of SOR (Stimulus-Organism-Response) paradigm. *Electron Mark.* (2023) 33:58. 10.1007/s12525-023-00674-z

[25] Ye Q Li Y Luo Y Pang Z. The impact of AI anchor anthropomorphism on users’ willingness to co-create value in tourism live-streaming contexts: the mediating role of social presence and the moderating role of perceived control. *Front Psychol*. (2026) 16:1724176. 10.3389/fpsyg.2025.1724176 41705167 PMC12908167

[26] Lu P Parrella JA Xu Z Kogut A. A scoping review of the literature examining consumer acceptance of upcycled foods. *Food Qual Prefer.* (2024) 114:105098. 10.1016/j.foodqual.2023.105098

[27] Ouro-Salim O Ramos HR Fanho AD. Upcycling as a circular strategy for food waste valorization in developing countries: a systematic review and the upgrade strategic framework Urban. *Agricul Reg Food Syst.* (2026) 11:e70042. 10.1002/uar2.70042

[28] Chu K DeGeorge D Diehn D Galatz A Garza J McGowan L et al. Upcycled vs sustainable: identifying consumer segments and recognition of sustainable and upcycled foods within the United States. *Foods.* (2025) 14:3508. 10.3390/foods14203508 41154043 PMC12562476

[29] D’Souza C Mancuso Tradenta JC Marimuthu M Fleming-Muñoz D O’Keefe S. The upcycled appetite: exploring intentions behind choosing food waste-based functional products and food neophobia. *Br Food J.* (2025) 127:760–80. 10.1108/bfj-04-2025-0517

[30] Zaman QU Cei L Rossetto L Thiene M Bedin B. Analysing the drivers of consumers’ attitudes and intention to buy upcycled food products: an application extending the theory of planned behaviour. *Br Food J.* (2026) 128:249–62. 10.1108/bfj-09-2025-1167

[31] Melios S Johnson H Grasso S. Sensory quality and regulatory aspects of upcycled foods: challenges and opportunities. *Food Res Int*. (2025) 199:115360. 10.1016/j.foodres.2024.115360 39658162

[32] Moshtaghian H Bolton K Rousta K. Upcycled food choice motives and their association with hesitancy towards consumption of this type of food: a Swedish study. *Br Food J*. (2024) 126:48–63. 10.1108/bfj-09-2022-0757

[33] Afari-Agyapong ME Jun K Jang M Yoon B. Exploring perceived values and purchase intentions for upcycled food: int1egrating survey analysis with text mining in South Korea. *Food Qual Prefer.* (2026) 136:105770. 10.1016/j.foodqual.2025.105770

[34] Zhang XY Chao CT Chen HS. Fostering sustainable food consumption: a theoretical framework for upcycled foods. *Front Psychol*. (2025) 16:1682850. 10.3389/fpsyg.2025.1682850 41229592 PMC12602388

[35] Cao Y Jang Y. Consumer perception of upcycled food labels. *Br Food J.* (2025) 127:3230–45. 10.1108/bfj-02-2025-0216

[36] Yu C Chang T. Evaluating the ethics of purchasing upcycled foods: insights into consumer intentions from an ethical decision-making perspective. *Curr Psychol*. (2025) 44:17040–55. 10.1007/s12144-025-08383-w

[37] Ye T Hwang Y Luo A Mattila AS. Promoting upcycled foods through message appeals: a moral signaling perspective. (2026) 38:1153–71. 10.1108/ijchm-06-2025-0825

[38] Wan A Jiang M. Can virtual influencers replace human influencers in live-streaming e-commerce? an exploratory study from practitioners’ and consumers’ Perspectives. *J Curr Issues Res Adv*. (2023) 44:332–72. 10.1080/10641734.2023.2224416

[39] Na R Hu A Zeng Q Ge R Hao R. Unveiling the relationship between virtual streamer characteristics and consumer purchase intention in live streaming commerce: Insights from source credibility perspective. *Electron Mark*. (2025) 35:68. 10.1007/s12525-025-00813-8

[40] Wen J Li XAI. Digital Human Responsiveness and Consumer Purchase Intention: The Mediating Role of Trust. *JTheorApp Electron Comm Res*. (2025) 20:246. 10.3390/jtaer20030246

[41] Zhu Z Hall CM Tao L Qin Z Li Y. When AI meets livestreaming: exploring the impact of virtual anchor on tourist travel intention. *J Theore App Electron Comm Res*. (2025) 20:239. 10.3390/jtaer20030239

[42] Gu Y Guo Z Hao J. Decoding virtual streamers: exploring emotional and cognitive drivers of consumer patronage intentions in live-streaming commerce. *Asia Pacific J Mark Logist*. (2026) 38:1061–76. 10.1108/apjml-02-2025-0238

[43] Qu Y Lo CKY Baek E. From humanoid to virtual humans: a systematic literature review of avatar marketing. *Int J Hum Comput Interact.* (2025) 41:12602–21. 10.1080/10447318.2025.2464889

[44] Lim RE Lee SY. “You are a virtual influencer!”: understanding the impact of origin disclosure and emotional narratives on parasocial relationships and virtual influencer credibility. *Comp Human Behav*. (2023) 148:107897. 10.1016/j.chb.2023.107897

[45] Yoo Y Yang HW Kim J Lim CH. The impact of virtual influencers’ anthropomorphism, attractiveness, credibility and sports knowledge on consumers’ information-sharing intentions. *Int J Sports Mark Sponsor.* (2025) 1–19. 10.1108/IJSMS-07-2024-0157

[46] Kim W Lee D Ham C. Human vs. artificial intelligence: the role of agent knowledge in consumer responses to ai influencers, moderated by interactivity and mediated by anthropomorphism. *J Adv*. (2026) 55:462–82. 10.1080/00913367.2025.2602465

[47] Zhang X Shi Y Li T Guan Y Cui X. How do virtual AI streamers influence viewers’ livestream shopping behavior? the effects of persuasive factors and the mediating role of arousal. *Inform Sys Front*. (2024) 26:1803–34. 10.1007/s10796-023-10425-2

[48] Brendl CM Sweldens S. Defining the stimulus in stimulus–response interventions: on the need to embrace theory and organism in stimulus–organism–response. *Con Psychol Rev.* (2024) 7:116–20. 10.1002/arcp.1098

[49] Li Y Chen X. Can corporate ESG practices promote consumers’ purchase intention of green food? *Front Sustain Food Syst.* (2025) 9:1622653. 10.3389/fsufs.2025.1622653

[50] Li Y Shan B. The influence mechanism of green advertising on consumers’ purchase intention for organic foods: the mediating roles of green perceived value and green trust. *Front Sustain Food Syst.* (2025) 9:1515792. 10.3389/fsufs.2025.1515792

[51] Lee S Sundar SS Lee J. From live streamer to influencer: credibility effects of authority. interactivity, and sponsorship. *Media Psychol*. (2025) 28:731–63. 10.1080/15213269.2024.2442991

[52] Chen J Luo J Zhou T. Research on determinants affecting users’ impulsive purchase intention in live streaming from the perspective of perceived live streamers’ ability. *Behav Sci.* (2024) 14:190. 10.3390/bs14030190 38540493 PMC10968506

[53] Oz E Oz F. Artificial intelligence-enabled ingredient substitution in food systems: a review and conceptual framework for sensory. functional, nutritional, and cultural optimization. *Foods*. (2025) 14:3919. 10.3390/foods14223919 41300077 PMC12651232

[54] Liao C Luo J Wang S Shen Y Lin S. How live-streaming commerce influences consumers’ impulse buying of near expiry-date food: implications for reducing food waste. *Front Sustain Food Syst.* (2025) 9:1570483. 10.3389/fsufs.2025.1570483

[55] Chen J Zeng Y Qiu X. Digital anchors vs. human anchors: a study of the effects of credibility endorsement and psychological distance on policy adoption intention. *Front Psychol*. (2025) 16:1650691. 10.3389/fpsyg.2025.1650691 41341728 PMC12670127

[56] Zhou X Li Z Huang X Hu X Li Z Shi J et al. Artificial intelligence-driven food safety and quality control. *J Food Measure Charac*. (2026) 20:4071–108. 10.1007/s11694-026-04088-1

[57] Heo H Lee S. Consumer information-seeking and cross-media campaigns: an interactive marketing perspective on multi-platform strategies and attitudes toward innovative products. *J Theor App Electron Comm Res*. (2025) 20:68. 10.3390/jtaer20020068

[58] Qiao K Sheng X Li ZAI-. Avatars versus human-avatars: a comparative study of user interaction behaviors in live streaming. *Int J Hum Comput Interact.* (2026) 42:9754–79. 10.1080/10447318.2025.2579785

[59] Woo E Kim YG. Consumer attitudes and buying behavior for green food products. *Br Food J.* (2019) 121:320–32. 10.1108/bfj-01-2018-0027

[60] Hussain Z Khan A Qureshi MA Bansal R Pruthi N. The impact of online reviews on sustainable product adoption in the food industry: a serial mediation effect of consumer trust and perceived value. *J Prom Manag*. (2025) 31:933–55. 10.1080/10496491.2025.2530056

[61] Wu Y Huang H. Influence of perceived value on consumers’ continuous purchase intention in live-streaming e-commerce—mediated by consumer trust. *Sustainability*. (2023) 15:4432. 10.3390/su15054432

[62] Dong C Wang X Luo Y Shi X. Virtual or human streamer? livestream channel strategy under AI empowerment. *Int J Prod Res.* (2026) 1–23. 10.1080/00207543.2026.2693740

[63] Lin Y Zou Y Wang X. Conditional trust pathways in live-streaming commerce: how consumer motivation influences responses to human and AI anchors. *Front Psychol*. (2026) 17:1797803. 10.3389/fpsyg.2026.1797803 42157987 PMC13180573

[64] Li C Huang F. The impact of virtual streamer anthropomorphism on consumer purchase intention: cognitive trust as a mediator. *Behav Sci*. (2024) 14:1228. 10.3390/bs14121228 39767370 PMC11672890

[65] Tian Z Chen F Cui D. The influence of e-commerce virtual streamer characteristics on consumers’ purchase intention: Based on a hybrid SEM-ANN approach. *Acta Psychol*. (2026) 266:106758. 10.1016/j.actpsy.2026.106758 41946143

[66] Yu T Teoh AP Bian Q Liao J Wang C. What drives purchase intention in live streaming e-commerce? *Int J Hum Comp Interact.* (2025) 41:14689–708. 10.1080/10447318.2025.2487721

[67] Nguyen NT Zhang Q Rehman SU Usman M Palmucci DN. Organic food and obesity: factors influencing actual purchase of organic food in COVID-19 pandemic with moderating role of organic food availability. *Br Food J*. (2023) 125:2190–216. 10.1108/bfj-02-2022-0120

[68] Slack NJ Singh G Ali J Lata R Mudaliar K Swamy Y. Influence of fast-food restaurant service quality and its dimensions on customer perceived value, satisfaction and behavioural intentions. *Br Food J.* (2021) 123:1324–44. 10.1108/bfj-09-2020-0771

[69] Jiang H Fan Z Wang J Liu S Lin W. The determinants of product trust in live streaming e-commerce: a hybrid method integrating SEM and fsQCA. *Asia Pac J Mark Logist.* (2025) 37:273–93. 10.1108/apjml-01-2024-0048

[70] Li Y Shan B. Exploring the role of health consciousness and environmental awareness in purchase intentions for green-packaged organic foods: an extended TPB model. *Front Nutr*. (2025) 12:1528016. 10.3389/fnut.2025.1528016 40110167 PMC11921780

[71] Watanabe EAdM Alfinito S Curvelo ICG Hamza KM. Perceived value, trust and purchase intention of organic food: a study with Brazilian consumers. *Br Food J.* (2020) 122:1070–84. 10.1108/bfj-05-2019-0363

[72] Howard MC Boudreaux M Oglesby M. Can harman’s single-factor test reliably distinguish between research designs? not in published management studies. *Eur J Work Organ Psychol.* (2024) 33:790–804. 10.1080/1359432x.2024.2393462

[73] Marcoulides KM Raykov T. Evaluation of variance inflation factors in regression models using latent variable modeling methods. *Educ Psychol Meas*. (2019) 79:874–82. 10.1177/0013164418817803 31488917 PMC6713981

[74] Fornell C Larcker DF. Evaluating structural equation models with unobservable variables and measurement error. *Jmark Res.* (1981) 18:39–50. 10.1177/002224378101800104

[75] Hair JF Black WC Babin BJ Anderson R. *EMultivariate Data Analysis.* 8th ed. Andover: Cengage (2019).

[76] Barrett P. Structural equation modelling: adjudging model fit. *Pers Individ Dif.* (2007) 42:815–24. 10.1016/j.paid.2006.09.018

[77] Cheung GW Lau RS. Testing mediation and suppression effects of latent variables: Bootstrapping with structural equation models. *Organ Res Methods.* (2008) 11:296–325.

[78] Liakos KG Athanasiadis V Bozinou E Lalas SI. Machine learning for quality control in the food industry: a review. *Foods*. (2025) 14:3424. 10.3390/foods14193424 41097592 PMC12523314

[79] Cook B Costa Leite J Rayner M Stoffel S van Rijn E Wollgast J. Consumer interaction with sustainability labelling on food products: a narrative literature review. *Nutrients*. (2023) 15:3837. 10.3390/nu15173837 37686869 PMC10489983

[80] Tang F. The more interactivity the better?. *Inf Technol Manag.* (2020) 21:179–89. 10.1007/s10799-020-00316-2

[81] Zeng W Ma Z Chen T. When and why do consumers resist virtual influencer endorsement? The role of product depth and advertising claim types. *Electron Comm Res*. (2026) 26:965–98. 10.1007/s10660-024-09903-9

[82] Huang W Tsai T Lai K Chen H. Analyzing consumer motivations and behaviors towards upcycled food from an environmental sustainability perspective. *Agriculture*. (2024) 14:1967. 10.3390/agriculture14111967

[83] Wu W Zhang A van Klinken RD Schrobback P Muller JM. Consumer trust in food and the food system: a critical review. *Foods*. (2021) 10:2490. 10.3390/foods10102490 34681539 PMC8536093

[84] Huh Y Xu Y Jeong E. Co-branding for sustainability: how consumer perceived benefits, risks, and quality assurance shape upcycled food acceptance. *Br Food J.* (2026) 128:386–406. 10.1108/bfj-04-2025-0446

[85] Ishigaki R Shreeves M Madhabika LN. Creating a circular economy by anthropomorphic product design. *IIM Kozhikode Soc Manag Rev*. (2026) 15:114–22. 10.1177/22779752251346636

